# Stem and Cancer Stem Cell Identities, Cellular Markers, Niche Environment and Response to Treatments to Unravel New Therapeutic Targets

**DOI:** 10.3390/biology10010025

**Published:** 2021-01-02

**Authors:** Jose R. Pineda, Iker Badiola, Gaskon Ibarretxe

**Affiliations:** 1 Department of Cell Biology and Histology, Faculty of Medicine and Nursing, University of the Basque Country (UPV/EHU), 48940 Leioa, Spain; iker.badiola@ehu.eus; 2 Achucarro Basque Center for Neuroscience Fundazioa, 48940 Leioa, Spain

Adult stem cells are a partially quiescent cell population responsible for natural cell renewal and are found in many different regions of the body, including the brain, teeth, bones, muscles, skin, and diverse epithelia, such as the epidermal or intestinal epithelium, among others. Interestingly, adult stem cell populations share all the instructions to grow and differentiate to any type of cell of the specific tissue they belong to. These normally quiescent stem cells can be activated on demand to replenish mature differentiated cell populations, a process which is driven mainly by signaling cues from its niche, thanks to the activation of their specific receptors in a timely and fine-tuned manner. One of the best examples of a very different niche regulation is the radically different turnover rate of intestinal stem cells with respect to neural stem cells. 

When someone evokes the word “cancer”, it can inspire fear and respect for a devastating and rampant disease, and similarly it could give the impression at first glance that it refers to a unique and singular pathology. However, years of research have made clear that there are at least as many cancers as tissues in the body, each with its unique characteristics endowed by the interactions with tissue-specific stromal cells [1]. According to the more than 40 year-old theory about the emergence of cancers, tumor cells are all derived from master cells called Cancer Stem Cells (CSCs), which would play a role similar to that of normal stem cells that are responsible for cell renewal of healthy tissues and organs, including self-renewing and differentiation capabilities [2,3,4]. Therefore, it is understandable that the research community has focused its efforts in the identification of these CSCs, in an attempt to cut cancer progression and metastasis at its roots. Even though CSCs have been postulated as principally responsible for cancer chemoresistance and recurrence, the interactions of these undifferentiated cancer cells with their surrounding stromal cells are also critical to understanding the difficulties of designing effective anticancer therapies [5].

Interestingly, tumor initiating CSCs, even when expressing the same cell surface receptors and being emplaced in the same niches of the normal stem cells, lose their internal control and grow indefinitely, becoming able to self-sustain differentiation towards endothelial-like vasculature cells and inducing vasculogenesis and angiogenesis to maintain blood and oxygen supply to the newly created and growing tumor mass [6]. Additionally, these cells acquire resistance to anoikis, allowing them to resist apoptotic signaling when they detach from their extracellular matrix and infiltrate surrounding tissue to spread and cause metastases. Surprisingly, despite the plethora of signals and events constantly changing in these scenarios, when we compare the molecular markers of a normal stem cell against its malign counterparts (e.g., neural stem cells against glioma stem-like cells in the brain, or intestinal stem cells against intestinal cancer stem-like cells in the gut), these are often not as different as could be expected. Moreover, many of the so far proposed CSC markers are also shared by a great deal of other normal stem cells in the body [7]. What then are the molecular marker criteria for defining a cell type as a CSC? Is there a way to target these cells selectively without compromising other stem cell types in the body? Those are some fundamental questions in modern oncology. Furthermore, as research evidence accumulates, it is becoming increasingly clear that some stem cell types can be quite resistant to transformation, even when they share more than a few characteristics with CSCs. One of the most intriguing cases is that of the dental pulp tissue, where no carcinogenesis or neoplasms have ever been reported to arise from the stem cells of the dental pulp [7]. This Special Issue, entitled “Stem and Cancer Stem Cells, Same Pathways for Different Malignancy”, addresses normal and cancer stem-like cell particularities, as well as the integration of the different signaling events and cell adaptations occurring after chemotherapy and radiotherapy treatments, highlighting their resistance to current treatments and oncogenic transformation.

This stem and cancer stem cell Special Issue welcomes new research advances in order to try to unravel the complexity of stem cell transformation and cancer stem cell resistance to current therapies.

## Data Availability

Not applicable.
